# Presaging Critical Residues in Protein interfaces-*Web Server* (PCRPi-*W*): A Web Server to Chart *Hot Spots* in Protein Interfaces

**DOI:** 10.1371/journal.pone.0012352

**Published:** 2010-08-23

**Authors:** Joan Segura Mora, Salam A. Assi, Narcis Fernandez-Fuentes

**Affiliations:** Section of Experimental Therapeutics, Leeds Institute of Molecular Medicine, University of Leeds, Leeds, United Kingdom; Monash University, Australia

## Abstract

**Background:**

It is well established that only a portion of residues that mediate protein-protein interactions (PPIs), the so-called *hot spot*, contributes the most to the total binding energy, and thus its identification is an important and relevant question that has clear applications in drug discovery and protein design. The experimental identification of hot spots is however a lengthy and costly process, and thus there is an interest in computational tools that can complement and guide experimental efforts.

**Principal Findings:**

Here, we present **P**resaging **C**ritical **R**esidues in **P**rotein **i**nterfaces-***W***eb server (http://www.bioinsilico.org/PCRPi), a web server that implements a recently described and highly accurate computational tool designed to predict critical residues in protein interfaces: PCRPi. PRCPi depends on the integration of structural, energetic, and evolutionary-based measures by using Bayesian Networks (BNs).

**Conclusions:**

PCRPi-*W* has been designed to provide an easy and convenient access to the broad scientific community. Predictions are readily available for download or presented in a web page that includes among other information links to relevant files, sequence information, and a Jmol applet to visualize and analyze the predictions in the context of the protein structure.

## Introduction

Cellular tasks require highly precise and regulated communication between proteins. Whether a protein is part of a metabolic pathway, an intermediate signalling effector, part of the transcription machinery, or a component of the cytoskeleton -just to mention some examples- requires proteins to act as complexes rather than as isolated units. Thus, protein-protein interactions (PPIs) are ubiquitous in Biology and therefore offer an enormous potential for the discovery of novel therapeutic agents able to modulate PPIs.

The analysis of protein complexes for which tertiary structure is known, has shown that protein interfaces are large, typically between 1500–2000 Ang^2^ [1], [2], involving many intermolecular contacts (10 to 30 side chains per protein on average), and that such surfaces are usually flat and lacking defining physicochemical traits. It is for that reason that the identification of small-molecules that can act as modulators of PPIs is widely regarded as a formidable goal. However, as recently reviewed by Wells and McLendon [3] (and references therein), exciting new data indicates that disruption of protein associations using small molecules is possible.

Part of the recent successes in the modulation of PPIs using small molecules has been possible by direct targeting of the important region, or *hot spot*, of the protein interface. The concept of hot spots in protein interfaces originates from the pioneering work of Clackson and Wells [4] that jointly with subsequent scientific works, have shown that most of binding energy in protein-protein associations can be ascribed to a small and complementary set of interfacial residues – a hot spot- surrounded by weaker interactions.

The experimental identification of hot spots in protein interfaces by Alanine scanning [5], Alanine shaving [6], or residue grafting [6], is a lengthy, labour-intensive, and costly process. Computational tools can be used to help and guide experimental efforts. We recently developed a novel computational tool: Presaging Critical Residues in Protein interfaces (PCRPi), that proved to be highly accurate and competitive with current computational methods [7]. In this paper, we present the implementation of the method as web application that will provide convenient and easy access to the method to the scientific community. The web application has been designed having in mind a wide range of potential users, thus it has a user-friendly and straightforward interface with a minimal number of tunable parameters. Predictions are readily available for download or presented in a web page that has a number of functionalities such as a Jmol applet to visualize and analyze the predictions in the context of the protein structure.

## Results and Discussion

### Submitting a task

**Figure 1:**
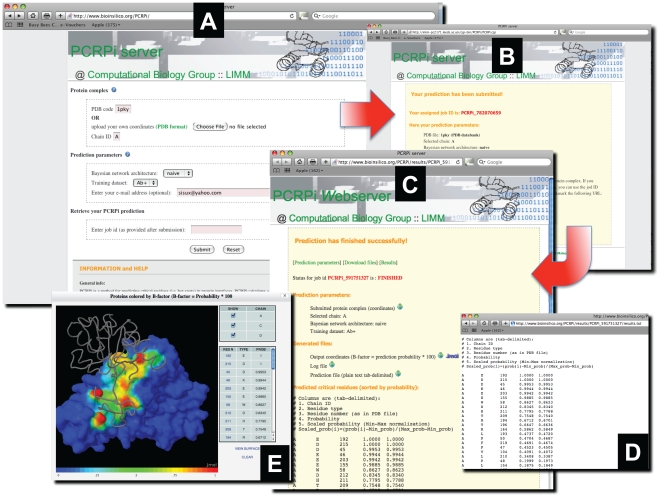
Several screenshots of PCRPi-*W*. The home web page of the server is the submission web page (A), where upon submission a temporary web page (B) reports an unique job identification code and a link to the results web page that users can bookmark to retrieve their results when available. The results web page (C) provides access to a number of links among them: a link to download the list of predicted hot spot residues (D) and a link to visualize the protein complex colored by prediction probabilities using a Jmol applet (E).

Running a prediction on PCRPI-*W* is a straightforward procedure. On the submission web page (Figure 1, panel A), users have to submit the coordinates of the protein complex of interest by either selecting it from a locally mirrored Protein Databank (PDB) database [8] typing the PDB code in a text box or uploading the coordinates (PDB format only); and select the chain identification code of the protein of interest. In advanced options, users can choose the type of BN and training set (see below).

Prior to prediction, structures undergo a set of quality checks. If atoms present alternative locations or rotamers, only the first occurring rotamer is kept. Also, if residues have insertion codes, the distance with neighboring residues is calculated and discarded if structurally equivalent. Side-chains with missing atoms are re-constructed using Scrwl 4.0 [9], an important step because energy calculations are highly affected by missing atoms. Finally, the length of proteins are checked and those shorter that 40 residues are discarded. As a result, a modified version of the original coordinate file, remediated coordinates file, is generated. This is the file used as input during the prediction and is downloadable from the result web page. Changes to the original coordinate (if any) are recorded in the log file (see below *Retrieving and visualizing results*).

**Figure 2:**
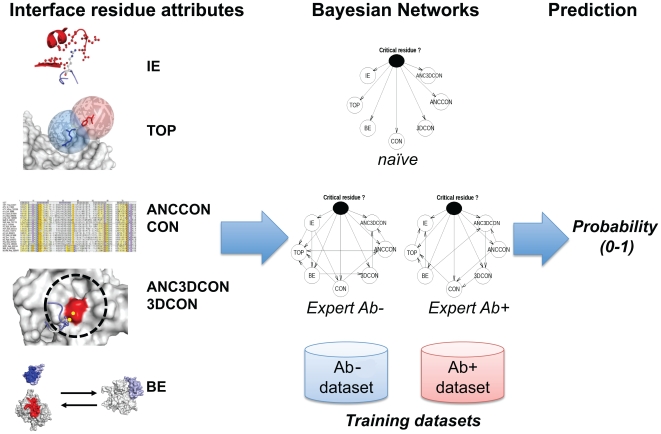
General overview of the prediction process. PCRPi combines seven different measures by using BNs and outputs a probability. The input variables are: IE, TOP, BE, CON, 3DCON, ANCCON, and ANC3DCON. There are two different training datasets: Ab+ and Ab−, and three different BNs: a naïve and two training dataset-specific experts BNs that can be invoked during the prediction. For more information regarding PCRPi method and input variables, refer to the original publication describing the method [7].

PCRPi-*W* features two types of BNs, a naïve and expert, that can be trained using two different datasets: Ab+ and Ab− (Figure 2). More information about the structure of the BNs and the composition of the training sets can be found in the help web page of the server or in the original publication describing the method [7]. By default, PCRPi-*W* run the prediction using a naïve version of the BN trained on the Ab+ dataset, although both, BNs type and training sets are tunable parameters and users can select the ones that adjust the best to their needs. If an e-mail address is given at time of the submission, user will be notified by e-mail once the job is finished including a hyperlink to the results web page (hyperlink also shown upon submission for bookmarking purposes; Figure 1, panel B). PCRPI-*W* assigns a unique job identifier for each submitted job (e.g. PCRPi_cA8r0nAz0). This job identifier can be used to check the status of the submission (i.e. in queue, running, finished) and to retrieve the results by typing it in the ‘Job ID’ field at the submission web page.

Jobs are handled by a queuing system and, if not competing jobs, typically take few minutes to be completed; larger protein complexes featuring large or multiple interfaces can take up to one hour. The most time consuming is the estimation of the binding free energy, which for large interfaces and protein complexes requires intensive and long computational times, and the sequence search and calculation of sequence profiles for evolutionary-based measures.

### Retrieving and visualizing results

PCRPI-*W* returns a list of interface residues sorted by probability (Figure 1 panel C and D) and several links to download files used or generated during the prediction. A successful prediction will generate the following files: a file that contains the original coordinates as uploaded by the user or as in the PDB; the remediated version of the coordinates file (see above *submitting a task*); a modified version of the input coordinates where the B-factor field has been substituted by a value that is equal to the prediction probability times 100 (facilitating analysis of predictions when using molecular visualization programs such as PyMOL [10]); a list of interface residues sorted by probability; a file detailing the atomic interaction of the interface residues as defined by CSU program [11] (atomic interactions can be also visualized in the context of the structure by using a Jmol (http://www.jmol.org) applet, see next); and a log file that records the entire prediction process and that can be examined if errors are reported.

Other elements that are shown in the results web page is the mapping of predictions on the protein sequence and a Jmol applet that allows the visualization of the structure of the complex and the mapping of the predictions. The Jmol applet includes a clickable list of protein chains and residues sorted by probability (Figure 1, panel E), and thus facilitate the process of visualization and selection of interface residues and predictions. Upon selection of a given residue, this will be highlighted in ball-and-stick representation and the atomic interactions with neighbouring residues will be shown.

### Possible bottlenecks

Occasionally, PCRPI-*W* may fail to provide a prediction. The main reason is usually when the coordinates file contains only one protein chain or if more that one, these do not interact, i.e. no atomic interactions between protein chains. In this case, interface(s) cannot be located and therefore the program fails. More rarely, there can be errors along the prediction process, e.g. problems during free energy calculations or errors when deriving evolutionary-based measures, e.g. PSI-BLAST [12] fails to find homologous sequences with significant E-values. As described above, a log file is available for users to download and examine to understand the reason(s) of reported error(s). In addition, users can contact the authors via e-mail for further support.

### Availability and Future Directions

PCRPi-W server is freely available upon registration to the scientific community at http://www.bioinsilico.org/PCRPi. Besides the option of submitting tasks to the server, users can browser an extensive documentation, have access to related resources available online, and download the benchmark and training datasets.

## Methods

### Prediction algorithm

Several are the features that characterize the residues that are part of a hot spot and these have been exploited in the past for prediction purposes. These features can be broadly grouped in three categories depending on nature of the data. Hot spots can be predicted by energy, structural, and evolutionary-based (e.g. sequence conservation) analysis. Although these features are useful, it was shown that, individually, cannot unambiguously define hot spots [13]. PCRPi [7] overcomes this limitation by combining a set of seven different measures that account for energetic, structural, and evolutionary-based information (Figure 2). Individual measures are combined into an unique probabilistic framework by using Bayesian Networks (BNs) [14], [15].

The performance of PCRPi was benchmarked in two independent datasets [7]. The first set was composed of 25 protein complexes summing up 636 interfaces residues, 300 of which were validated as critical or non-critical residues by experimental means and available in the scientific literature. The second dataset was the protein complex formed by HRAS and a VH domain of an Fv antibody [16]. Under both scenarios PCRPi delivered highly accurate and consistent predictions. Moreover, in a head-to-head comparison with other available computational tools using the same test set, PCRPi predictions were superior in terms of precision, recall, and F1-scores (Table 1).

**Table 1:** Comparison of different methods for the prediction of critical residues in protein interfaces using a BID derived dataset as described in Tuncbag et al. [18].

Method	Precision (P)	Recall (R)	F1 score
PCRPi^a^	0.79	0.64	0.71
FoldX^b^	0.75	0.36	0.49
Robetta-Ala^c^	0.63	0.57	0.60
KFC^c^	0.51	0.36	0.42
KFC-A^c^	0.53	0.48	0.51
LDA^c^	0.72	0.57	0.64
Tuncbag et al. [18] c	0.73	0.59	0.65

a Predictions were performed using PCRPi [7] with an expert BN trained in a Ab+ dataset that does not include the crystal structure of the c2 fragment of streptococcal protein G in complex with the Fc domain of human Ig (PDB code 1fcc).

b Values were obtained running FoldX [19] with default parameters and a ddG_binding_ cut-off of 2.0 Kcal.mol^−1^ (i.e. residues were considered critical if upon mutation to Ala, predicted ddG_binding_≥2.0 Kcal.mol^−1^).

c Precision, recall, and F1 score values taken from Tuncbag et al. [18].

### Design, implementation and use of PCRPi-*W*

PCRPI-*W* is implemented on an Apache server running on a Red Hat® enterprise linux-based operating system. The server is interfaced with a CGI Perl and Javascript coded web interface. PCRPI-*W* modules and accessory scripts are coded in Perl, Fortran, and C++ respectively. Databases required by the server, namely, PDB [8] and NCBI non-redundant (NR) protein sequence database [17], are locally mirrored and weekly updated. All the queries are submitted to a queuing system that submits the tasks to a computer farm. Results are displayed in HTML format and send to the user by e-mail containing a hyperlink to the results web page.
